# Monotypic plasma cell interstitial nephritis as the only clinical manifestation in a patient with previously undiagnosed indolent multiple myeloma: A case report

**DOI:** 10.1097/MD.0000000000004391

**Published:** 2016-08-07

**Authors:** Philippe Attias, Anissa Moktefi, Marie Matignon, Jehan Dupuis, Céline Debiais-Delpech, Philippe Grimbert, Philippe Lang, Vincent Audard

**Affiliations:** a Assistance Publique-Hôpitaux de Paris (AP-HP), Service de Néphrologie et Transplantation, Institut Francilien de Recherche en Néphrologie et Transplantation (IFRNT), Centre de Référence Maladie Rare Syndrome Néphrotique Idiopathique, Groupe Hospitalier Henri–Mondor/Albert-Chenevier; b AP-HP, Département de Pathologie, Groupe Hospitalier Henri-Mondor/Albert-Chenevier; c Université Paris-Est-Créteil (UPEC), Département Hospitalo-Universitaire (DHU) Virus-Immunité-Cancer (VIC), Institut Mondor de Recherche Biomédicale (IMRB), Equipe 21, INSERM U 955; d AP-HP, Unité d’Hémopathies Lymphoïdes, Groupe Hospitalier Henri-Mondor/Albert-Chenevier; e UPEC, DHU VIC, IMRB, Equipe 9, INSERM U 955, Créteil; f Centre de Référence des Amyloses Primitives et des Maladies de Dépôts d’Immunoglobulines Monoclonales; g Département de Pathologie, Centre Hospitalier Universitaire de Poitiers, Poitiers, France.

**Keywords:** chemotherapy, interstitial nephritis, kidney, monoclonal gammopathy, plasma cells

## Abstract

**Introduction:**

Predominantly monotypic plasma cell infiltrates are an uncommon renal finding in patients with malignant lymphoplasmacytic proliferation.

**Case presentation:**

We report the case of a 52-year-old man with chronic kidney disease and significant proteinuria associated with a monoclonal immunoglobulin spike (IgGκ). Kidney biopsy revealed the presence of atypical multinucleated CD138^+^ plasma cells with voluminous nuclei stained exclusively with a κ antibody. Electron microscopy showed mesangial and segmental parietal electron-dense, nonorganized hyaline deposits without immunogold labeling for the κ light chain. The bone marrow aspirate revealed 6% of apparently mature plasmocytes without dystrophy. We therefore concluded that the patient had an indolent multiple myeloma with specific renal involvement in the form of malignant monotypic interstitial plasmacytic infiltration. We initiated a specific chemotherapy regimen including bortezomib–cyclophosphamide–dexamethasone. After 4 months of follow-up, creatinine levels had improved slightly and free κ light-chain levels had decreased significantly within the normal range.

**Conclusion:**

This case highlights the need to consider neoplastic interstitial plasma cell infiltration systematically in patients diagnosed with an apparently benign monoclonal gammopathy and to consider adaptation of the chemotherapy regimen, to improve renal function.

## 1 Introduction

The spectrum of renal diseases associated with monoclonal gammopathy is wide and continually expanding. In most cases, monoclonal immunoglobulin (Ig)-related kidney disorders occur in patients with malignant lymphoplasmacytic proliferation, such as multiple myeloma (MM), Waldenström macroglobulinemia, chronic lymphocytic leukemia, and non-Hodgkin B-cell lymphoma.^[1]^ Due to their intrinsic physicochemical properties, monoclonal Ig can induce glomerular and tubular damage, which can be classified into 2 categories based on the nature of the Ig deposits observed on electron microscopy (organized or nonorganized deposits).^[1,2]^ In addition to renal disorders directly related to Ig deposition, malignant interstitial infiltrates may also occur in patients displaying a monoclonal Ig-secreting cell proliferation. In 1 study, lymphomatous infiltration of the interstitium was the main pathological feature observed in 40% of patients undergoing renal biopsy in a context of non-Hodgkin lymphoma.^[3]^ Three of 14 patients with Waldenström macroglobulinemia also had isolated neoplastic lymphoplasmacytic infiltration of the interstitium.^[4]^ In patients with chronic lymphocytic leukemia or small lymphocytic lymphoma, monotypic interstitial infiltration may be isolated or associated with specific glomerular involvement.^[5]^ Renal lymphomatous infiltration may be the first clinical manifestation of underlying B-cell proliferation in some patients.^[6]^ The exclusive interstitial proliferation of monotypic plasma cells is a very rare finding in patients with MM. This renal condition is generally considered to be a renal plasmacytoma with typical clinical manifestations and radiological features occurring preferentially in patients with a diagnosis of high-mass MM.^[7]^ We report here a case of neoplastic interstitial infiltrate composed almost exclusively of neoplastic monotypic plasma cells in a patient with an apparently benign monoclonal gammopathy.

## 2 Clinical report

A 52-year-old man of Caribbean origin was referred to our nephrology department for the investigation of renal failure. He had a history of hypertension treated with nicardipine (100 mg/d) and urapidil (60 mg twice daily) and had undergone cholecystectomy in 2006 for acute cholecystitis. At the time of cholecystectomy, serum creatinine concentration was 1.24 mg/dL (estimated glomerular filtration rate [eGFR], 83 mL/min/1.73 m^2^ according to the Chronic Kidney Disease Epidemiology Collaboration equation). Findings at admission included a blood pressure of 125/90 mm Hg, heart rate of 61 beats/min, and no fever. Physical examination revealed no orthostatic hypotension, organomegaly, macroglossia, palpably enlarged lymph nodes, and peripheral sensitive or motor neuropathies. Laboratory investigation yielded an increase of serum creatinine level, at 2.43 mg/dL (eGFR [Chronic Kidney Disease Epidemiology Collaboration], 34 mL/min/1.73 m^2^). We observed marked proteinuria (urine protein/creatinine ratio of 217 mg/mmol) associated with leukocyturia (13/mm^3^) without microscopic hematuria on urinary sediment analysis. The 24-hour urine sample (2.3 g protein/d) contained mostly albumin, with 0.12 g of free κ light chains. Electrolyte concentrations, including serum calcium concentration, were within the normal range and liver function test results were normal. Blood tests showed an absence of anemia (hemoglobin level of 124 g/L) and white blood cell and platelet counts were in the normal range (6 × 10^9^/L and 236 × 10^9^/L, respectively). Serological screening for hepatitis was negative. Immunological tests for antineutrophil cytoplasm, and antinuclear and anti-DNA antibodies were negative. Serum complement levels were within the normal range. Serum protein electrophoresis showed total protein concentration to be normal (65 g/L), with a moderately low albumin concentration (34.7 g/L). On immunoelectrophoresis, we detected a monoclonal IgGκ spike (7.9 g/L) associated with low levels of γ-globulin (3.5 g/L). Immunonephelometric assays confirmed the presence of free κ light chains at a concentration of 114 mg/L (normal range: 6.7–22.4 mg/L) with a κ/λ ratio of 7.08 (normal range: 0.31–1.56 mg/L). Urine immunoelectrophoresis was positive for the κ light chain and albuminuria. Renal ultrasonography and abdominal computed tomography scan findings were unremarkable, and both kidneys were normal in size, with no focal lesions. Bone marrow aspiration showed normally maturing hematopoietic progenitors and apparently mature nondystrophic plasma cells accounted for 6% of all cells. No osteolytic lesions were observed on bone x-ray and whole-body magnetic resonance imaging failed to show any focal bone lesions.

**Figure 1:**
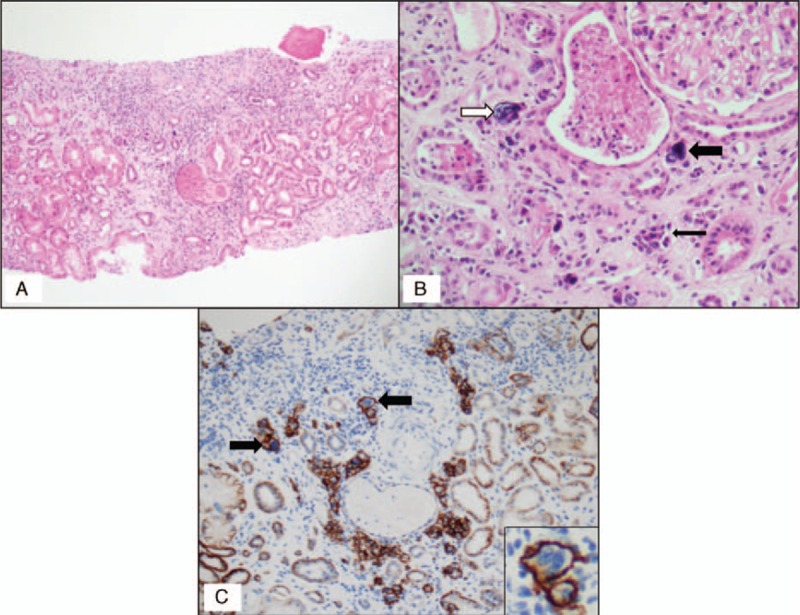
(A) Light microscopy revealed chronic tubulointerstitial nephritis with heterogeneous fibrosis and a cell infiltrate (hematoxylin and eosin staining, ×100). (B) The cell infiltrate consisted mostly of clustered plasma cells with an amphophilic cytoplasm and eccentric nuclei (thin black arrow; hematoxylin and eosin staining, ×200). Presence of atypical multinucleated plasma cells (thick black arrow) and scattered interstitial “monster” cells with voluminous, irregular, hyperchromatic nuclei and a high nuclear/cytoplasmic ratio (white arrow; hematoxylin and eosin staining, ×400). (C) Immunohistochemistry (anti-CD138 ×200, monoclonal mouse anti-human, clone MI15, code M 7228, DAKO, Glostrup, Denmark, dilution 1:100). The atypical cells (arrows and right corner) were CD138^+^ immature plasma cells.

**Figure 2:**
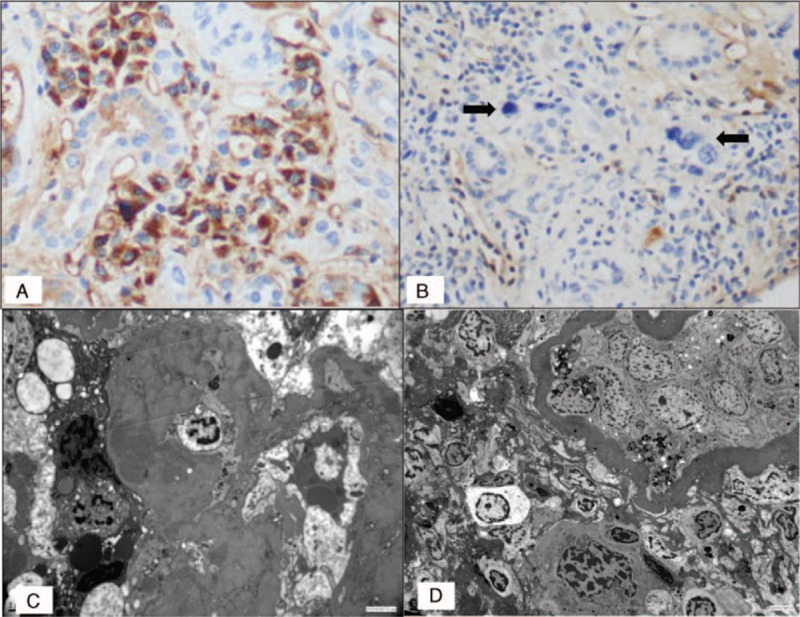
Anti-κ immunoglobulin light-chain (A) and anti-λ immunoglobulin light-chain (B) immunohistochemistry revealed bright positive staining for monotypic κ aggregates in the cytoplasm of plasma cells (×400). Immunohistochemical staining for λ immunoglobulin light chain was negative in atypical cells (B, arrows). (C) Electron microscopy magnification showing a glomerular capillary loop with nonorganized osmiophilic deposits (×6000). (D) Electron microscopy magnification confirmed the presence of interstitial polylobated, pleomorphic plasma cells with prominent nucleoli, abundant cytoplasm, and high nuclear–cytoplasmic ratio (×3000).

A percutaneous renal biopsy was performed, due to the occurrence of renal failure associated with significant proteinuria in a context of IgGκ monoclonal gammopathy. The renal biopsy specimen consisted of renal cortex with 11 glomeruli including 4 obsolescent glomeruli. Four glomeruli displayed focal and segmental glomerulosclerosis (FSGS) lesions, whereas the others presented moderate, patchy mesangium expansion without cell proliferation. The tubulointerstitial areas displayed diffuse mononuclear cell infiltration within extensive fibrosis (50%) (Fig. 1A). The cell infiltrate consisted almost entirely of atypical binucleated or multinucleated plasma cells with voluminous, irregular, hyperchromatic nuclei (Fig. 1B). Focal nonspecific thickening of the tubular basement membranes was observed, with flattening and atrophy of the tubular epithelium but no typical myeloma casts. No tubulitis lesions were observed. Mild atherosclerotic changes were observed in the arterial vessels. In cellular infiltrate, immunostaining demonstrated the large predominance of CD138^+^ cells (Fig. 1C). Atypical plasma cells were associated with nontumoral interstitial inflammation involving rare T (CD3^+^) and B (CD20^+^) cells (data not shown). Immunofluorescence studies revealed an absence of heavy-chain and/or light-chain Ig deposits on the tubular basement membrane and glomerular structures, and highlighted polytypic hyaline casts. Congo-Red staining was negative. Immunohistochemistry study showed that only atypical infiltrating plasma cells were positive for κ light chains (Fig. 2A and B). Immunohistochemical staining for the IgG4 antibody was negative, ruling out a diagnosis of IgG4-related kidney disease (data not shown). Electron microscopy showed mesangial and segmental parietal electron-dense, nonorganized hyaline deposits and interstitial polylobated, pleomorphic plasma cell infiltrate (Fig. 2C and D). Immunogold labeling excluded κ light-chain deposits in the glomeruli (data not shown). Given the ethnic origin of the patient, we investigated whether the FSGS lesions could be related to an *APOL1* risk variant genotype. Genotyping of the *APOL1* gene showed the patient to be heterozygous for the G1 and G2 *APOL1* risk alleles. Positron emission tomography was performed, due to the diagnosis of renal acute interstitial neoplastic plasma cell infiltration. No highly hypermetabolic lesions were detected. The bone marrow trephine biopsy was infiltrated by monotypic plasma cells. We therefore diagnosed neoplastic monotypic plasma cell interstitial nephritis associated with chronic kidney disease (defined as a permanent [lasting at least 3 months] decrease in eGFR to <60 mL/min/1.73 m^2^ according to the modification of diet in renal disease formula) in this patient with indolent MM.

We obtained patient consent and prescribed an induction chemotherapy regimen consisting of a bortezomib–cyclophosphamide–dexamethasone protocol. After 4 cycles of the bortezomib–cyclophosphamide–dexamethasone regimen, renal function had improved slightly (creatinine level = 2.08 mg/dL) but proteinuria had not decreased (urine protein/creatinine ratio of 228 mg/mmol) despite a significant decrease in κ light-chain levels (22.4 mg/L) and the normalization of κ/λ ratio.

## 3 Discussion

Infiltrate consisting predominantly or exclusively of plasma cells is an uncommon renal finding in patients with acute interstitial nephritis. The typical hallmarks of renal parenchyma injury associated with IgG4-related kidney diseases are fibrosis and massive infiltration of the renal interstitium with lymphocytes and IgG4-positive plasma cells.^[8]^ Melica et al described 2 patients with human immunodeficiency virus infection and acute interstitial nephritis related to diffuse infiltration of the kidney interstitium, predominantly with CD138^+^ plasma cells.^[9]^ In these 2 renal disorders, the plasma cells were positive for both κ and λ light chains, excluding malignant plasmacytic infiltration. In our case, the presence of voluminous atypical CD138^+^ plasma cells and absence of IgG4 deposits strongly suggests that the infiltrate was malignant. The definitive diagnosis of tumoral plasma cell infiltration was based on immunohistochemistry findings showing that the atypical cells were positive for the κ light chain only. Strikingly, specific renal involvement was not associated with the significant clonal proliferation of malignant plasma cells in the bone marrow. The potential value of chemotherapy in this context remains to be clearly demonstrated.

MM is associated with a broad spectrum of renal lesions, mostly caused by the deposition of monoclonal Ig in the renal parenchyma.^[1]^ The spectrum of tubulointerstitial lesions associated with MM usually includes kidney injury directly resulting from monoclonal Ig deposits (light-chain deposition diseases, amyloid light chain amyloidosis, cast nephropathy, and proximal tubulopathies). A recent study showed that proximal tubulopathies were a frequent renal biopsy finding for these patients (46% of cases have lesions related to plasma cell dyscrasia).^[10]^ In our case, electron microscopy findings were not suggestive of monoclonal light-chain tubulopathies. Moreover, 22 of 70 patients undergoing renal biopsy for suspected myeloma cast nephropathy diagnosis were found to display >25% cortical interstitial inflammation, but none of these patients presented interstitial infiltration with neoplastic plasma cells.^[11]^ These data strongly suggest that specific tumoral infiltration of the renal interstitial area with monotypic plasma cells is rare in patients with MM.

Our patient displayed significant proteinuria, with FSGS lesions on renal biopsy examination, but immunofluorescence and immunogold labeling studies detected no monotypic κ deposits in glomeruli, ruling out a diagnosis of glomerular disease related to the monoclonal Ig component. By contrast to what has been reported for intraglomerular lymphomatous infiltration in patients with aggressive lymphoproliferative disorders,^[6]^ the renal biopsy specimen showed no infiltration of CD138^+^ plasma cells into the glomeruli. The main cause of FSGS in our patient may be related to the *APOL1* polymorphism known to be associated with a higher risk of FSGS.^[12]^ Nevertheless, Dingli et al described a cohort of 13 patients with apparent “idiopathic” FSGS and plasma cell disorders.^[13]^ A close pathophysiological relationship between these 2 entities was suggested, because of the significant improvement in proteinuria observed on chemotherapy, together with the remission of plasma cell disorders in 4 patients with MM.^[13]^ In our patient, the main cause of the FSGS lesions remains hypothetical.

The clinical, pathological, radiological, and therapeutic management of renal extramedullary plasmacytoma have been extensively reviewed by Zhang et al.^[7]^ Renal plasmacytoma, classically observed in cases of high-mass MM, is usually associated with severe renal injury and with radiological findings mimicking solitary renal tumors.^[7]^ The features of the lymphomatous infiltration typically associated with non-Hodgkin lymphoma include acute kidney injury and enlarged kidneys, with or without nodular defects on radiological examination.^[6]^ The radiological findings for our patient were unremarkable, and renal function remained stable during follow-up.

We describe here the first case, to our knowledge, of direct plasma cell infiltration of the renal interstitium in a patient with indolent MM without any other symptoms of MM than renal failure. There is compelling evidence to suggest that renal disease involving the deposition of an entire Ig or of its components may occur in patients diagnosed with monoclonal gammopathy of undetermined significance. Thus, the notion of a “monoclonal gammopathy of renal significance” (MGRS) has recently emerged as a new renal entity characterized by Ig-related kidney disease in patients not meeting the criteria for MM or lymphoproliferative disorders.^[14,15]^ Definitive confirmation of the diagnosis of MGRS by renal biopsy is important, because specific treatment (mostly with chemotherapy-based regimens) can sometimes stabilize or improve renal injury. Cohen et al studied 49 patients with monoclonal Ig deposition disease. They found that 38 patients diagnosed with MGRS were treated with chemotherapy regimens including bortezomib plus dexamethasone (and additional treatment for 18 patients).^[16]^ Hematological and renal responses were detected in some of these patients, highlighting the potential utility of chemotherapy in patients with MGRS. Our case, characterized by indolent MM revealed by exclusive neoplastic interstitial infiltration without concomitant monoclonal heavy-chain and/or free light-chain deposits on tubular and/or glomerular structures, is very unusual. The renal findings for this patient led us to initiate specific chemotherapy to decrease the proliferation of renal plasma cells. After 4 cycles of chemotherapy, no significant improvement in renal function and proteinuria was observed. However, renal biopsy revealed the presence of initially severe chronic tubulointerstitial fibrosis and FSGS lesions not classically amenable to chemotherapy. The κ/λ ratio normalized after chemotherapy, but no significant decrease in proteinuria was observed. These findings suggest that the initial marked proteinuria was mostly due to FSGS lesions.

In conclusion, the possibility of predominantly tumoral plasma cell infiltration of the interstitium should be systematically considered in patients with an indolent MM, and should be differentiated from other classical glomerular and/or tubular lesions observed in patients with MGRS spectrum conditions. The potential utility of chemotherapy remains unclear, but, given the rarity of this renal entity, such treatment should be systematically considered, to at least stabilize renal function.
